# Deficient Purposeful Use of Forepaws in Female Mice Modelling Rett Syndrome

**DOI:** 10.1155/2015/326184

**Published:** 2015-06-22

**Authors:** Bianca De Filippis, Mattia Musto, Luisa Altabella, Emilia Romano, Rossella Canese, Giovanni Laviola

**Affiliations:** ^1^Behavioural Neuroscience Section, Department of Cell Biology and Neurosciences, Istituto Superiore di Sanità, Viale Regina Elena 299, 00161 Rome, Italy; ^2^Molecular and Cellular Imaging Section, Department of Cell Biology and Neurosciences, Istituto Superiore di Sanità, Viale Regina Elena 299, 00161 Rome, Italy

## Abstract

Rett syndrome (RTT) is a rare neurodevelopmental disorder, characterized by severe behavioural and physiological symptoms. Mutations in the methyl CpG binding protein 2 gene (*MECP2*) cause more than 95% of classic cases. Motor abnormalities represent a significant part of the spectrum of RTT symptoms. In the present study we investigated motor coordination and fine motor skill domains in MeCP2-308 female mice, a validated RTT model. This was complemented by the* in vivo* magnetic resonance spectroscopy (MRS) analysis of metabolic profile in behaviourally relevant brain areas. MeCP2-308 heterozygous female mice (Het, 10-12 months of age) were impaired in tasks validated for the assessment of purposeful and coordinated forepaw use (*Morag test* and* Capellini handling task*). A fine-grain analysis of spontaneous behaviour in the home-cage also revealed an abnormal handling pattern when interacting with the nesting material, reduced motivation to explore the environment, and increased time devoted to feeding in Het mice. The brain MRS evaluation highlighted decreased levels of bioenergetic metabolites in the striatal area in Het mice compared to controls. Present results confirm behavioural and brain alterations previously reported in MeCP2-308 males and identify novel endpoints on which the efficacy of innovative therapeutic strategies for RTT may be tested.

## 1. Introduction

Rett syndrome (RTT) is a rare neurodevelopmental disorder that primarily affects females with an incidence of 1 in 10.000 births. Mutations in the gene encoding for the methyl-CpG-binding protein 2 (MECP2) have been identified in more than 95% of classic RTT cases [1, 2]. No cure is available at the moment except for symptomatic treatments [3].

RTT syndrome is characterized by an apparently normal perinatal development until 6–18 months of age, when patients start losing their acquired skills in the motor, social, and cognitive domains [4, 5]. At the end of the regression period, a wide variety of symptoms is manifested, which include loss of spoken language, kyphosis/scoliosis, and respiratory impairment [6]. The discovery of a monogenic origin for classical RTT led to the creation of several mouse lines bearing different mutations on the* MeCP2* gene [3, 7]. These models recapitulate RTT-like symptomatology, including social, cognitive, and respiratory alterations, thus representing valuable tools for the RTT preclinical research [3].

Motor abnormalities represent a significant part of the spectrum of symptoms affecting RTT patients and are the cause of a marked decreasing in the quality of their life [8]. The most common ones are the stereotyped hand movements and the gait disturbances, which occur in almost every patient [9]. Motor coordination alterations like apraxia and ataxia as well as cases of dystonia and axial hypotonia are also frequent [8].

Despite the wide spectrum and high relevance of motor dysfunction in RTT, the analysis of motor impairments in RTT mouse models is still far from complete. Available reports in the major RTT mouse lines have explored motor coordination, motor learning, and locomotor profile in both male and female MeCP2-deficient mice (see, e.g., [10–14]). In a recent paper, the gait analysis in a MeCP2 mouse model also revealed that alterations in measures such as stride, coordination, and balance are evident before the onset of other overt phenotypic changes [15].

The present study has specific relevance in this framework, since it provides a first in-depth investigation of purposeful use of forepaws in the MeCP2-308 mouse model [16]. This mutation is associated with a milder phenotype, a delayed onset of symptoms, and a longer life-span compared with knockout (ko) mouse models [17]. To increase the translational value of the study, heterozygous (+/−, Het) MeCP2-308 female mice and wild-type controls (+/+, wt) were used.

For the sake of completeness, the investigation of motor function was integrated by the ethological scoring of the mouse spontaneous behaviour in the home-cage, to control for general locomotor activity levels and time budgeting of different behavioural domains, which may also unveil subtle changes in motivation towards specific activities.

A methodological add in the present study was the inclusion of the analysis of metabolites content in selected behaviourally relevant brain areas in MeCP2-308 female mice by ^1^H magnetic resonance spectroscopy (^1^H MRS). We aimed, by means of this non-invasive imaging tool that allows the* in vivo* characterization of the brain metabolism, to determine whether the mild pathologic processes which were uncovered in RTT patients [18, 19] and in MeCP2-308 males [20] have equivalents in MeCP2-308 female mice.

## 2. Methods

### 2.1. Subjects

The experimental subjects were fully symptomatic (10–12 months of age) MeCP2-308 Het female mice [B6.129S-MeCP2tm1Heto/J, stock number: 005439; backcrossed to C57BL/6J mice for at least 12 generations from the Jackson Laboratories (USA)] [16, 17] and age-matched wild-type (wt) littermate controls (13 wt and 15 Het). At weaning, mice were housed in sex-matched groups of 2-3 in polycarbonate transparent cages (33 × 13 × 14 cm) with sawdust bedding and kept on a 12 h reversed light-dark schedule (lights off at 8:00 am). Temperature was maintained at 21 ± 1°C and relative humidity at 60 ± 10%. Animals were provided* ad libitum* with a complete pellet diet (Altromin, Germany).

All procedures were carried out in accordance with the European Communities Council Directive (2010/63/UE) and formally approved by Italian Ministry of Health.

### 2.2. Genotyping

DNA was prepared from a small tail-tip biopsy taken at 21–28 days of age. The* MeCP2* alleles were identified by PCR using two sets of primers. Primer set 1 (5′ primer: 5′-CAC CAC AGA AGT ACT ATG ATC-3′ and 3′ primer: 5′-CTA GGT AAG AGC TCT TGT TGA-3′) yields a product of 180 bp identifying the wild-type allele. Primer set 2 (5′ primer same as for primer set 1 and 3′ primer: 5′-ATG CTG ACA AGC TTT CTT CTA-3′) yields a product of apparent size 260 bp identifying the null allele. PCR products were electrophoresed through a 2% NuSieve 3 : 1 agarose gel (Cambrex Bio Science, Rockland, ME, USA) containing 0.5 *μ*g/mL ethidium bromide and examined under UV light.

### 2.3. Behavioural Testing

All mice were experimentally naïve at the start of the test battery. The order of testing was as follows:* Dowel test, nest building, spontaneous behaviour in home-cage, Capellini handling test, and MoRaG task.* The* spontaneous behaviour with nesting material* was assessed in a second cohort of mice (11 wt and 12 Het). All mice were isolated 24 h before starting the test battery and remained isolated till the end. There was a minimum of 24 h between each test. All behavioural testing took place during the dark phase of the day between 9.00 am and 6.00 pm. Body weight of the animals was recorded before and after the behavioural test battery. The execution of the experiments and the analyses of the data as well as the randomization were all carried out by different experimenters, to reduce any possible bias.

#### 2.3.1. Dowel Test

To confirm previous data in MeCP2-308 hemizygous male mice [17], body balance and coordination were evaluated with the Dowel test. The hardwood round dowel used was 9.0 mm in diameter and 35 cm long. The dowel was mounted horizontally 50 cm above a 5 cm deep bedding of sawdust. At the beginning of the testing, each mouse was placed in the middle of the dowel so that the length of its body was parallel to the dowel. Each mouse was tested for 4 consecutive trials. An intertrial interval of at least 10 minutes was used. Latency to fall from the dowel into a cage of bedding was recorded. If mice were able to walk across the dowel and off of the dowel, they received the maximum score of 30 seconds.

#### 2.3.2. Capellini Handling Test

Previous characterizations of mouse eating behaviour have revealed that they use coordinated forepaw movements to manipulate food pieces. The Capellini handling test was thus carried out to evaluate purposeful hand use and coordination of mouth and forelimbs. Testing occurred as previously described, with minor modifications [21], in a custom-made chamber with the floor made of transparent Plexiglas to optimize the video recording of paws movements and subsequent video analyses. The test consisted of two consecutive trials of variable duration, with one piece of pasta eaten per each trial (2.5 cm in length). The day before testing, the experimental mice were habituated to the experimental apparatus during a 10-min long session, to prevent the anxiety deriving from the exposure to a novel context. Moreover, to overcome neophobic responses during the test, for three consecutive days before starting the test, animals were exposed to short lengths of uncooked capellini pasta in their home-cages. To increase the motivation to eat, mice were primed to perform the task by restricting access to food for up to 16 h before the task.

The testing sessions were videotaped and subsequently scored by a trained observer blind to the genotype of the mice, with the Noldus Observer Software. For fine-grain analysis of forepaws movements, slow motion video playback was used (50% of real-time). For each testing session, we recorded the* total duration of each trial*. The effective* time taken to consume the whole piece of pasta* was calculated by subtracting, from the total time, any pause in the interaction with the pasta. The* mean time to eat* for the 2 pieces of pasta was calculated for each mouse and considered an index of proficiency of the coordinated use of mouth and paws.

To identify any alterations in the handling pattern of RTT mice, the following variables were also scored:* one paw* (the mouse held the piece of pasta with either the left or the right forepaw),* grasp paw* (the mouse held the piece of pasta with both forepaws, using the right or left as “guide paw”),* symmetrical hold* (the mouse held the piece of pasta with both forepaws using a symmetrical position). The* grasp paw* and* one paw* hold positions were subsequently summed up and analysed within the “*asymmetrical holds*” category.

#### 2.3.3. Mouse Reaching and Grasping Test (MoRaG)

To further explore skilled aspects of motor function in RTT mice, the Mouse Reaching and Grasping test (MoRaG), a paradigm specifically developed for the quantitative and qualitative assessment of* reaching* and* grasping* motor parameters in the mouse, was applied.

The MoRaG apparatus consisted of a custom-made Plexiglas chamber (10.5 cm high by 7.5 cm deep by 6 cm wide) built as previously described [22]. A feeding platform was located at 5.5 cm from the floor on the outside front wall of the chamber. To force the mouse to use forelimbs to collect the food reward and to prevent the nose-poking behaviour, the platform was accessible through a 6 cm wide opening only.

Prior to training, mice were familiarized with the food rewards (a small piece of pasta of approximately 0.1 g). Throughout the testing period, mice were maintained on a restricted feeding schedule at not less than 85% of their free-feeding weight.

Mice were subjected to a training schedule that consisted of one daily trial. Each trial began when the pellet was delivered to the feeding platform and ended when the mouse had collected 30 food rewards or 30 minutes had elapsed. Each mouse was thus subjected to a variable number of trials and the test was considered to be successfully completed when 30 food rewards had been collected within a 30-minute session. The testing sessions were videotaped and subsequently scored by a trained observer blind to the genotype of the mice, with the Noldus Observer Software. As no differences were found as for the number of trials needed to reach the criterion (to collect 30 rewards within 30 minutes) between the experimental groups (5 ± 1 daily trials for both genotypes), the performance of the mice during the last successful trial was compared. Mice that did not perform any attempt to retrieve the food reward during the first two habituation sessions were excluded (4 MeCP2-308 Het and 3 wt females). The following parameters were recorded: time needed to collect 30 rewards* (total duration of the test)*,* number of reaching errors,* and* number of grasping errors.*

#### 2.3.4. Home-Cage Circadian Activity

The assessment of daily spontaneous activity in the home-cage was carried out by means of an automatic device using small passive infrared sensors positioned on the top of each cage (ACTIVISCOPE system). Mice were individually housed and assigned to a continuous monitoring of activity [17]. The access of the authorized personnel to the animal room was not restricted during the circadian locomotor activity recording and it followed the routine schedule. The system operated continuously for seven days. The 24 h profile of activity was obtained by averaging seven days of continuous registration. The sensors (20 Hz) detected any movement of mice with a frequency of 20 events per second. Data were recorded by an IBM computer with dedicated software. No movements were detected by the sensors when mice were sleeping, were inactive, or performed moderate self-grooming. Scores were obtained during 1 h intervals and expressed as counts per minute (cpm). The position of cages in the rack was such that mice of each genotype were equally distributed in rows and columns.

#### 2.3.5. Spontaneous Behaviour in Home-Cage

MeCP2-308 female mice are characterized by reduced locomotor activity, as highlighted by the automatic monitoring of activity in the home-cages. With the aim of uncovering whether this general profile may be due to either motor or motivational alterations, we decided to monitor the spontaneous behaviour in the home-cages (standard cages, 33 × 13 × 14 cm) of individually housed mice during 3 time intervals per day (11.00-12.00; 14.00-15.00; 18.00-19-00) for 3 consecutive days.

The scoring was carried out by a trained observer, blind to mouse genotype, by instantaneous sampling at an interval of 2 min during three 1 h sessions, for a total of 15 samples per session for each mouse. The daily profile of activity was obtained by averaging 4 days of registration. During each instantaneous scoring sample, the presentation of the following behavioural items was recorded:* inactivity* (complete absence of movements including small movements of head, ears, or vibrissae),* wall rearing* (body in vertical position with forepaws placed on the walls of the cage),* rearing* (body in vertical position),* self-grooming* (mouth or paws on body),* face-washing* (paws on face),* digging*,* burrowing*,* bar holding*,* sniffing*,* feeding,* and* drinking*. % frequency of each behaviour on the total number of observations per each time interval was subsequently calculated for statistical analysis.

#### 2.3.6. Nest Building

The performance in a nest-material manipulation task was evaluated as previously described in MeCP2-308 male mice [20, 23]. Briefly, one piece of filter paper (10 cm × 12 cm) was provided to each singly housed mouse 3 h after the lights switched off. After 24, 48, 72 h, and 7 days, quality of the nests was scored by a trained observer, blind to mouse genotype. Nest quality was measured using the following four-point qualitative scale [24]: 0: nest material untouched; 1: nest material nearly untouched; 2: nest material scattered, no clear shape evident; 3: nest of intermediate quality; 4: nest round and well built.

#### 2.3.7. Spontaneous Behaviour with Nest Material

To unravel whether the worse quality of the nests built by MeCP2-308 mice was due to motivational or motor coordination deficits in the integrated use of mouth and forelimbs, the spontaneous behaviour in the home-cage of the experimental mice was scored in the presence of nesting material. The nest material was introduced into the cage during the dark/active phase of the light/dark cycle, 3 h after the lights switched off. The interaction with the nesting material was scored by instantaneous sampling at an interval of 2 min during two 30-min sessions, starting at 1 and 4 hs after the introduction of the nesting material into the cage (15 samples per session for each mouse). The behaviour was scored directly in the colony room. Live scoring was preferred over off-line observation to clearly discriminate subtle behavioural patterns [25].

The following interactions with the nesting material were recorded: interacting with the material with the mouth only (*gnawing*), interacting with the paws only, and handling the material simultaneously with the paws and the mouth. The following indexes were subsequently calculated for statistical analyses: % observations interacting with material over the total number of observations (*% time interacting with nesting material*), % time interacting with the mouth on total time interacting (*% use of the mouth only*), % time interacting with the paws on total time interacting (*% use of the paws only*), and % time interacting simultaneously with the paws and the mouth on total time interacting (*% coordinated use of mouth and forelimbs*).

### 2.4. Magnetic Resonance Spectroscopy Experiments

*In vivo* ^1^H MRS was applied as previously described [20]. Briefly, animals were anaesthetised with 3.5% sevoflurane in oxygen 2 L/min (Sevoflo, Abbott SpA, Latina, Italy) within an induction chamber. Experiments were conducted on a 4.7 T Varian Inova animal system (Varian Inc., Palo Alto, CA, USA), equipped with actively shielded gradient system (max 200 mT/m, 12 cm bore size). A 6-cm diameter volume coil was used for transmission in combination with an electronically decoupled receive-only surface coil (Rapid Biomedical, Rimpar, Germany). In particular, the metabolic feature of the striatum and hippocampus areas (shown in Figure 5(a)) were studied, since one major aim in the actual MRS study in females was to verify the possible overlap with previous data in MeCP2-308 hemizygous male mice [20]. Importantly, these brain areas play a well-recognised role in the control of integrated motor function and cognitive capacities, two domains highly impacted by MeCP2 mutations. A quantitative ^1^H MRS protocol, including water T2 measurements, was applied [26]. Spectra were analysed using LCModel [27].

### 2.5. Statistical Analysis

Preliminary sample size estimation was carried out considering the two-tail Student *t*-test for independent groups using the following values, based on the results of previous studies: (i) standard deviation homogeneous among groups = 15.4; (ii) minimum difference between control and treatment group means that we would like to find out as significant delta = 20 (i.e., delta = 1.30 sigma, corresponding to the 80% reference interval of control subjects); (iii) type I error probability alfa = 0.05 and power 1 − beta = 0.80 (conventional values). The sample size resulting from this calculation was 11 subjects per group.

Parametric data were analyzed using *t*-tests or with repeated measures ANOVA if there was a within-subjects factor. The alpha level was set to 5%. Where data violated the assumptions of normality or equality of variance, the Mann-Whitney *U* tests were performed for simple group comparisons with no within-subjects factors.* Post hoc* comparisons were performed by Tukey HSD. The Levene test was applied to confirm the equality of variance. The presence of outliers was verified with Grubbs' test.

## 3. Results

### 3.1. Body Weight

The analysis of body weight revealed that fully symptomatic MeCP2-308 female mice weighted in general slightly, but significantly, more than wt controls (wt = 24.13 ± 0.25 g; Het = 26.13 ± 0.42 g;* main effect of genotype*: *F*_(1,26)_ = 11.18; *P* = 0.002).

### 3.2. Dowel Test

We found that MeCP2-308 Het female mice fell from the dowel significantly earlier than wt controls (Het: 6.10 ± 1.26 s; wt: 14.24 ± 1.53 s;* main effect of genotype*: *F*_(1,26)_ = 10.09; *P* = 0.004). An impairment in motor coordination and balance in Het female mice was thus confirmed in this test [17, 20]. No significant differences were found between trials.

### 3.3. Capellini Handling Test

**Figure 1:**
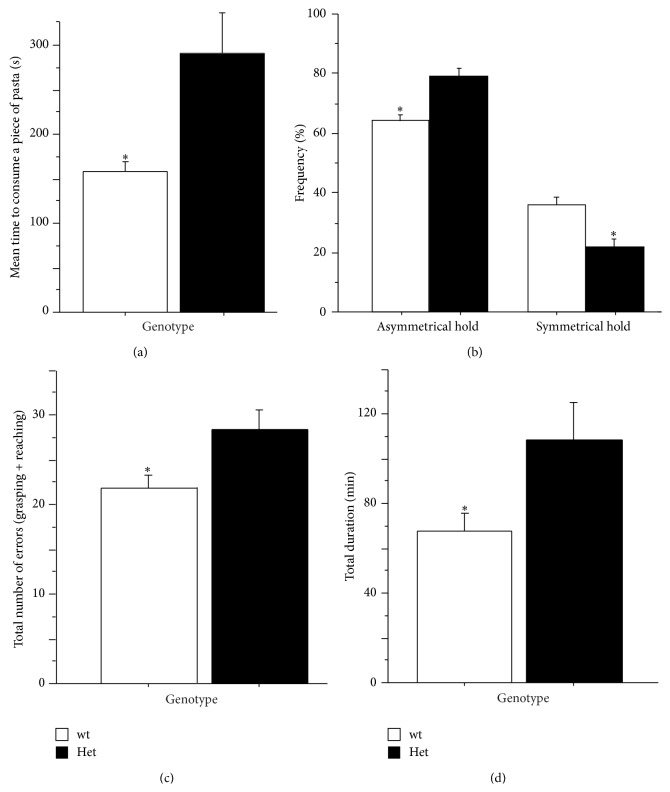
*Skilled and purposeful forepaw use and coordination in the Morag test and the Capellini handling task.* In the Capellini handling task, MeCP2-308 Het female mice (Het) needed more time to consume a piece of pasta compared to wild-type mice (wt) (a). Het mice also showed an atypical handling pattern compared to wt (b), as demonstrated by a higher number of* asymmetrical holds* (sum of* grasp paw* and* one paw* hold positions) and a significantly lower number of* symmetrical holds* (the mouse held the piece of pasta with both forepaws using a symmetrical position). In the Morag test, Het needed more time to collect 30 rewards (*total duration*) (d) and performed a higher number of* total number of errors* (sum of* incorrect reachings* and* incorrect graspings*) compared to wt controls (c). Data are expressed as mean ± SEM. ^*∗*^ *P* < 0.05.

To assess forepaw dexterity and evaluate coordination of hand use of RTT mice, we analysed the ability of MeCP2-308 Het female mice to handle a piece of pasta (uncooked capellini) [21]. We did not find any difference in the* total duration of the trial sessions* between genotypes. However, when we considered the effective time taken by RTT mice to consume the whole piece of pasta, we found that it resulted significantly higher (about 50% more) than that of wt subjects (*main effect of genotype: F*_(1,17)_ = 4.70; *P* = 0.044; Figure 1(a)). In this line, mean number of holds per piece tended to be higher in RTT mice, compared to wt controls (Het: 19.39 ± 3.50; wt: 10.70 ± 1.22;* main effect of genotype: F*_(1,17)_ = 3.86; *P* = 0.066). This profile suggests a marked reduced efficiency in the ability to handle the piece of pasta.

Unlike rats, mice use both paws for guiding, holding, or advancing the pasta during eating [21]. As a consequence, the typical handling pattern in the mouse consists of both symmetrical and asymmetrical holds. The fine-grain video analysis we carried out uncovered an atypical handling pattern in MeCP2-deficient mice. In particular, the relative use of* asymmetrical holds* was significantly higher in RTT compared to wt controls (*main effect of genotype*: *F*_(1,17)_ = 15.57; *P* = 0.001; Figure 1(b)), whereas* symmetrical holds* were lower (*main effect of genotype*: *F*_(1,17)_ = 15.57; *P* = 0.001; Figure 1(b)). Such a general profile suggests impairment in the coordinated use of the mouth and forepaws in RTT mice.

### 3.4. MoRaG Test

To further explore fine motor skills in RTT mice, a test specifically developed for the quantitative and qualitative assessment of reaching and grasping motor parameters in the mouse was adopted, the Mouse Reaching and Grasping (MoRaG) task [22]. We found that time needed to collect 30 rewards was significantly higher in MeCP2-deficient mice (*t*_(19)_ = −2.46; *P* = 0.024; Figure 1(d)). This was accompanied by a higher number of* total errors* (*incorrect reaches* +* incorrect grasps*) (*t*_(19)_ = −2.19; *P* = 0.041; Figure 1(c)), thus confirming an impairment of RTT mice in skillful catching of the food pellet (see also supplementary videos S1 and S2 in Supplementary Material available online at http://dx.doi.org/10.1155/2015/326184).

### 3.5. Home-Cage Circadian Activity

**Figure 2:**
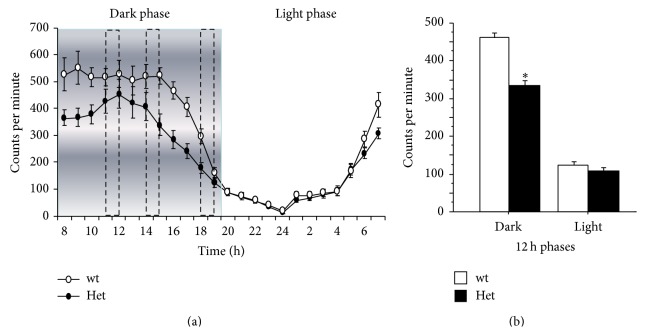
*Daily spontaneous locomotor activity in the home-cage*. MeCP2-308 heterozygous female mice (Het, *N* = 13) showed decreased home-cage spontaneous circadian locomotor activity compared to age-matched wild-type controls (wt, *N* = 15) during the dark/active phase of the L/D cycle. Mice were individually housed in the home-cages and locomotor activity was automatically recorded. The infrared sensors detected any movement of mice with a frequency of 20 events per second. Scores were obtained as counts per minute expressed during 1 h periods, and the 24 h profile of daily activity was obtained by averaging 7 days of continuous registration. (a) Representation of the 24 h activity profile, Het (black circles) and wt mice (white circles). The area evidenced in gray represents the dark/active phase of the day and the dash lines indicate the intervals during which the spontaneous behaviours in home-cages were scored by the experimenter (see Figure 3). (b) Representation of the 12 h activity profile. Data are expressed as mean ± SEM. ^*∗*^ *P* < 0.05.

Spontaneous locomotor activity and circadian rhythmicity of individually housed mice from both genotypes were recorded during a 24 h period, along seven consecutive days. As expected, mice from both genotypes exhibited a general increment of activity during the dark-phase of the L/D cycle (*main effect of the 12 h phase*: *F*_(1,19)_ = 168.36; *P* < 0.001; Figure 2(a)). MeCP2-308 Het female mice were characterized by a general reduction in locomotor activity compared to wt controls, as confirmed by a main effect of genotype (*F*_(1,19)_ = 7.13; *P* = 0.015; Figure 2(a)).* Post hoc* comparisons on the* genotype by 12 h phase interaction* (*F*_(1,19)_ = 6.56; *P* = 0.019) confirmed that MeCP2-deficient mice were significantly less active than wt subjects during the dark/active phase of the L/D cycle (*P* < 0.01; Figure 2(b)).

### 3.6. Spontaneous Behaviour in Home-Cage

**Figure 3:**
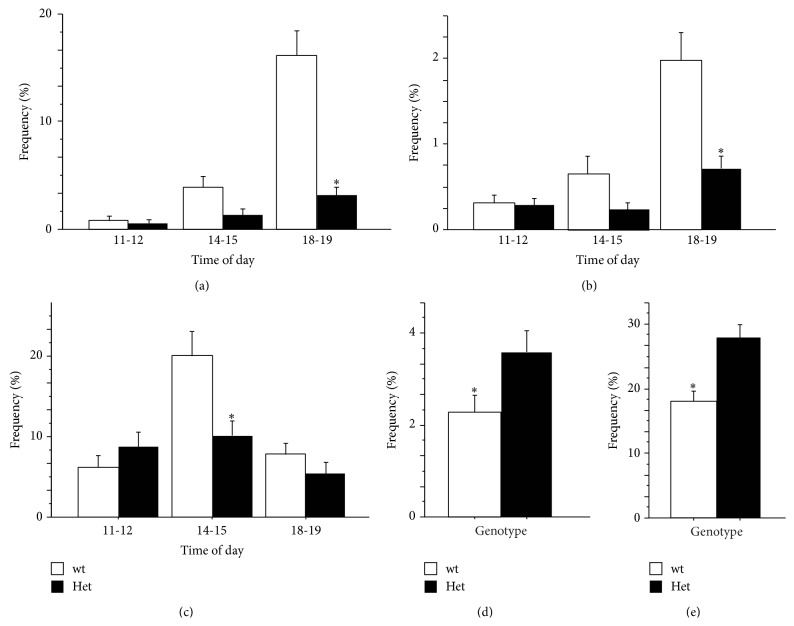
*Spontaneous behavioural profile in the home-cages.* The analysis of spontaneous behaviour in the home-cages revealed that MeCP2-308 heterozygous female mice (Het) show decreased cage-directed exploratory behaviours in the home-cages compared to wild-type controls (wt).* Burrowing* (a) and* digging* (b) and* bar holding* episodes (c) were significantly less frequent in Het mice. Het mice spent more time* drinking* (d) and* eating* (e) compared to wt. The spontaneous behaviour in home-cages of individually housed mice was scored 3 times per day (11.00-12.00; 14.00-15.00; 18.00-19-00; highlighted by dashed lines in Figure 2). Each session consisted of 15 observations. The profile of daily activity was obtained by averaging 3 consecutive days of data collection. Data are expressed as % frequency ± SEM. ^*∗*^ *P* < 0.05.

The data collected from the 3 days of direct observation of the mouse spontaneous behaviour in the home-cages evidenced a characteristic behavioural profile in MeCP2-308 Het female mice. Indeed, consistently with the reduced locomotor activity uncovered by the automatic monitoring in the home-cages (Figures 3(a) and 3(b)), we evidenced a reduced time devoted to general exploratory behaviour by MeCP2-deficient mice. In particular, when compared to wt subjects, RTT mice showed significantly lower levels of* burrowing* and* digging* during all the three sessions of observation (*main effect of genotype*: burrowing: *F*_(1,26)_ = 11.44; *P* = 0.002; digging: *F*_(1,26)_ = 5.25; *P* = 0.030; Figures 3(a) and 3(b)). Such a profile appeared particularly marked on the third interval (18-19 pm), in the presence of a strong increase in these behaviours by wt mice (*P* < 0.01, after* post hoc comparison on the genotype by time of day interaction*. Burrowing: *F*_(2,52)_ = 19.71; *P* < 0.001; digging: *F*_(2,52)_ = 4.62; *P* = 0.014) (Figures 3(a) and 3(b)). A significant reduction in* bar holding* behaviour was also evident in MeCP2-deficient mice compared to wt controls during the middle session, the time point at which wt mice showed a peak (*P* < 0.01, after* post hoc comparison on the genotype by time interval interaction: F*_(2,52)_ = 4.12; *P* = 0.001; Figure 3(c)). Of note, the reported reduction in environment-directed behaviours by RTT mice was complemented by a significant increase in time spent in self-maintenance activities, such as drinking and eating compared to wt subjects (*main effect of genotype*: drinking: *F*_(1,26)_ = 4.18; *P* = 0.051; eating: *F*_(1,26)_ = 6.58; *P* = 0.016) (Figures 3(d) and 3(e)). This is consistent with the increased body weight we uncovered in RTT mice compared to wt controls. No differences were found between the 3 different sessions of observation.

### 3.7. Nest Building Quality Score

**Figure 4:**
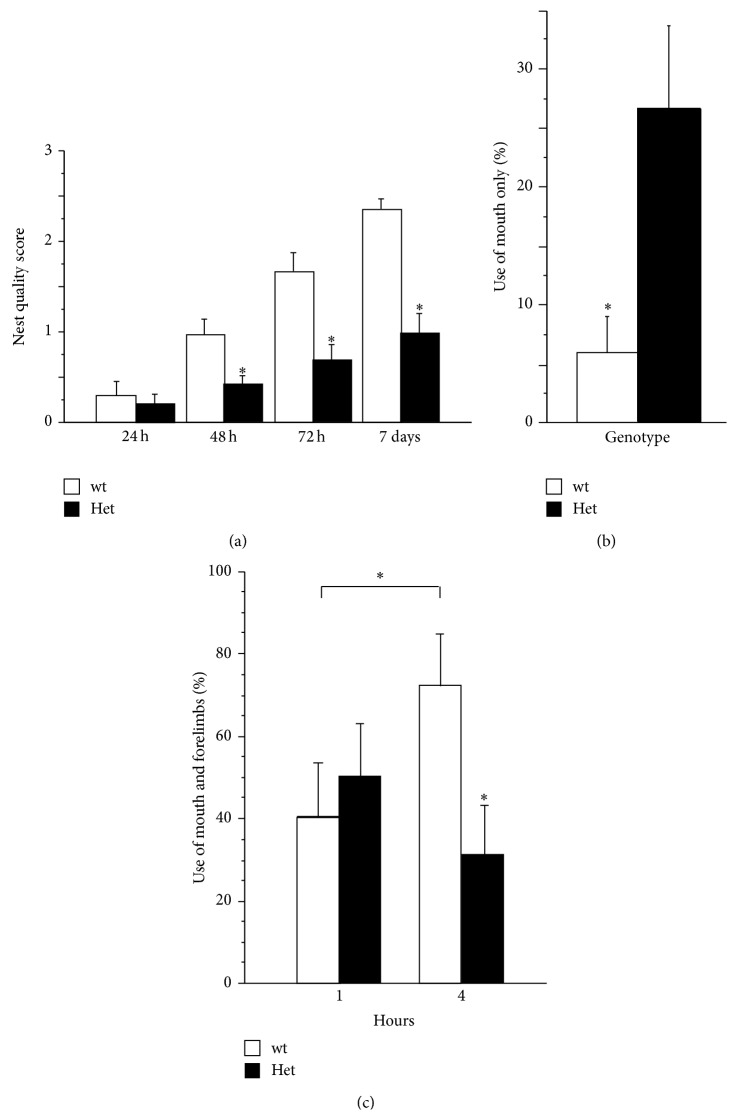
*Nest building abilities in RTT female mice*. (a) MeCP2-308 female mice (Het, *N* = 13) built nests of significantly poorer quality compared to wild-typecontrols (wt, *N* = 15). The quality of the nest was evaluated at 2 h, 48 h, 72 h, and 7 days after the introduction of nesting material into the cage (scores: 0 = bad quality; 4 = high quality). (b) The analysis of spontaneous behaviour in the home-cages revealed that Het mice more frequently interacted with the material with their mouth only, a condition which was rarely observed in wt controls. (c) The simultaneous and coordinated use of the mouth and the forepaws needed to build a high quality nest was deficient in Het mice compared to wt littermates. Data are expressed as mean ± SEM. ^*∗*^ *P* < 0.05.

As expected, the quality of the nests improved with time, with the highest score being generally evident after 7 days from the introduction of the nesting material into the cages (*repeated measures*: *F*_(3,78)_ = 10.46; *P* < 0.001; Figure 4(a)). Even though such improvement was evident in both genotypes, in keeping with previous studies in males [20, 23], we found that, compared to wt subjects, the nesting-material manipulation scores were generally lower in MeCP2-308 Het female mice (*genotype by repeated measures interaction*: *F*_(3,78)_ = 10.47; *P* < 0.001).

### 3.8. Spontaneous Behaviour with the Nest Material

When the spontaneous behaviour in the home-cages was monitored in the presence of the nesting material, we found that RTT mice spent a similar proportion of time interacting with the nesting material compared to wt littermates. An equal level of motivation toward this activity is thus suggested (*main effect of genotype: F* < 1; *P* > 0.40; data not shown). In keeping with the hypothesis that the poor quality of nest building in RTT mice may be due to a deficit in the integrated use of mouth and forelimbs [17, 23] we found that RTT mice more frequently interacted with the material with their mouth only (*main effect of genotype: F*_(1,21)_ = 8.15; *P* = 0.009; Figure 4(b)), a modality of interaction with the nesting material which was rarely observed in wt controls (less than 10%, Figure 4(b)). Moreover, RTT mice spent a much lower proportion of time handling the paper using simultaneously the mouth and the paws compared to wt littermates. As shown in Figure 4(c), this genotype-related difference emerged quite evident during the second scoring session, when wt mice showed an 80% preference for the coordinated use of mouth and forepaws while handling the material (*P* < 0.05 after* post hoc* comparison on the* genotype by treatment by repeated measures interaction: F*_(1,21)_ = 4.73; *P* = 0.041).

### 3.9. Brain Metabolic Profile

**Figure 5:**
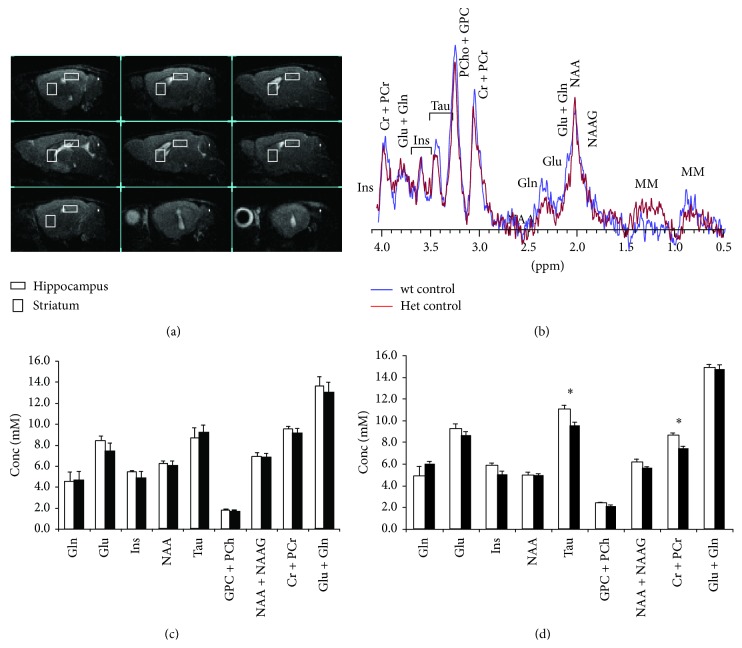
*Brain metabolic profile.* (a) Examples of* in vivo* sagittal T2-weighted spin-echo MR images (TR/TEeff = 3000/60 ms, slice thickness 0.8 mm, ns = 4, 13 slices, FOV = 25 × 25 mm^2^, and matrix 256 × 256). (b) Example of* in vivo* ^1^H MR spectra (PRESS, TR/TE = 4000/23 ms, NS = 256) acquired from a voxel positioned in the striatum (indicated by the white rectangles in (a)) of a wild-type (wt) and MeCP2-308 female mice (Het). (c) and (d) Metabolite levels obtained from* in vivo* ^1^H spectra in the hippocampus (c) and the striatum (d), obtained by using a quantitative protocol (which uses water as internal standard) and LCModel fitting program. Metabolite assignments: Cr, creatine; Gln, glutamine; Glu, glutamate; GPC, glycerophosphocholine; Ins, myo-inositol; NAA, nacetylaspartate; NAAG, n-acetylaspartylglutamate; PCho, phosphocholine; PCr, phosphocreatine; Tau, taurine. Data are expressed as mean ± SEM. ^*∗*^ *P* < 0.05.

The* in vivo* quantitative ^1^H MRS analyses detected genotype-associated differences in the spectra acquired in the striatum area of wt and MeCP2-308 female mice (Figure 5(d)). In keeping with a previous report in male mice [20], we found that in this brain area MeCP2-308 Het female mice show a lower concentration of taurine (Tau) and total creatine (Cr + PCr), metabolites involved in bioenergetics (Tau: *t*_(19)_ = 2.72; *P* = 0.013; Cr + PCr: *t*_(19)_ = 3.22; *P* = 0.045; Figure 5(d)). Differently from MeCP2-308 males [20], however, no differences were found as for the astrocytic marker myo-inositol. No genotype-related differences were evidenced in the hippocampal area (Figure 5(c)). Water T2 analyses confirmed that no changes occurred in the T2s within the groups, confirming that the alterations observed in the NMR signals were due to changes in the levels of metabolites.

Notably, the quantitative approach for brain metabolite determination we have adopted in the present study has been previously validated in several animal models of developmental disorders [20, 28–30]. This approach, which provides a robust measure of metabolite level, allowed us to evidence genotype differences in brain creatine content in the present study. This result further highlights the need for caution in the use of creatine-based ratio measurements of metabolites in developmental disorders.

## 4. Discussion

The present study provides clear evidence that female mice modelling RTT are significantly impaired in tasks validated for the assessment of purposeful and coordinated forepaw use. A fine-grain analysis of spontaneous behaviour in the home-cage also allowed us to unveil a general reduced motivation towards the exploration of the cage environment and increased time devoted to feeding behaviour. Further, the evaluation of metabolic brain profile by* in vivo* MRS investigation highlighted decreased levels of metabolites involved in bioenergetics specifically in the striatal area of MeCP2-308 female mice. Of note, such detailed neurobehavioural characterization was carried out in Het female subjects, the genetic and hormonal milieu which more closely recapitulates that of RTT patients [7].

A major finding of the present study is the demonstration that RTT female mice are characterized by a severe impairment in the purposeful use of forepaws, a motor ability known to be highly compromised in RTT patients [9].

The* Morag task* specifically evaluates the ability of the mouse to reach and grasp a food reward [22]. Such a paradigm has been applied to investigate models of brain damage and disease, mainly pertaining to stroke and Parkinson's disease [31]. Interestingly, such a skilled motor ability in the mouse has been found to be impaired by focal motor cortex stroke [32]. We found that RTT mice are highly impaired in this task, as demonstrated by the increased number of both incorrect* reachings* and incorrect* graspings* they performed.

This analysis was complemented by the* Capellini handling test*, a paradigm specifically developed to evaluate the ability of mice to manipulate food pieces by performing coordinated forepaw movements [21]. We found that MeCP2-308 mice needed significantly more time than wt mice to consume the piece of pasta, and these increased times were accompanied by an atypical handling pattern and by a reduced capacity of RTT mice to perform and maintain a correct holding position. MeCP2-deficient mice did in fact less frequently engage the* symmetrical hold*, a position which requires coordinated forepaws movements [21]. Atypical handling patterns have been already reported in previous studies in rats with a pharmacologically induced depletion of dopaminergic neurons [33], highlighting the role of basal ganglia in performing fine coordinated forepaws movements.

The demonstration that MeCP2-308 Het female mice show impairment in nest building ability of a magnitude comparable to that previously reported in MeCP2 hemizygous males [20] is also provided in the present study. Previous studies have suggested that such alterations in RTT mice may be due to an inability to handle and properly manipulate the nesting material [23]. Our results lie in the same direction. The monitoring of the spontaneous behaviour exhibited by RTT mice in the presence of the nesting material does in fact allow us to exclude a possible contribution of decreased motivation, which may indirectly affect the mouse performance and thus the quality of the nests built. By contrast, we uncovered an atypical handling pattern of the nesting material in RTT mice, characterized by a preferential use of the mouth for handling purposes.

Present results are consistent with the alterations RTT female mice showed in the* MoRaG* and the* Capellini task* and suggest that fine-motor movements and coordination of mouth and paws are specifically impaired in RTT mice and might account for their deficient nest building abilities.

As a whole, these results highlight a deficit in fine finalized movements of forepaws for the first time in a validated RTT mouse model [21, 22]. Together with previous studies demonstrating a general impairment in motor skills [3, 15], our data provide clear evidence that MeCP2-308 mice recapitulate the variety of motor symptoms that highly compromise the quality of life of RTT patients.

The evaluation of fine-motor skills and coordination in RTT mice was integrated in the present study by the characterization of the circadian locomotor profile and the time budgeting of general activities in the home-cage. The presence of a hypolocomotor profile was confirmed in RTT female mice [11, 34]. Moreover we report here that such profile was accompanied by a reduced frequency of environment-directed behaviours, such as* burrowing*,* digging,* and* bar holding* episodes. Taken together these data point to a decreased environment-directed attention (cage exploration) as the main component of the general hypoactivity profile by RTT mice. These data also support previous findings in Het* Mecp2* ^*Stop/+*^ females [11]. Interestingly, however, such a reduced overall activity does not seem to have affected the motivation of the RTT mice to perform behavioural tests. In particular, we found that RTT mice were motivated as far as wt mice to retrieve and eat the food, despite their lower performance, in both the Morag task and the Capellini test. Further, RTT mice spent a similar proportion of time interacting with the nesting material compared to wt littermates.

In keeping with previous evidence [11], we also found that RTT female mice were characterized by increased body weight and devoted increased time to spontaneous eating and drinking activities in the home-cage. This is consistent with previous literature findings demonstrating a role for MeCP2 in the regulation of binge eating disorders and body weight regulation [35, 36]. Moreover, present data strongly argue against a reduced interest of RTT mice toward appetitive stimuli. This is particularly interesting as both the paradigms we adopted to assess paw dexterity in RTT mice (the* MoRaG* and the* Capellini task*) were appetitively motivated. Thus, these results further corroborate the less efficient handling capacity of RTT mice.

As a whole, our data provide support to the increasing number of studies demonstrating that MeCP2 Het female mice represent valuable models for RTT [3, 11, 13, 14]. MeCP2-308 female mice were in fact found to recapitulate some motor alterations previously observed in male mice and several novel ones pertaining to forepaws use were identified [16, 17, 23]. Moreover, we here demonstrate that MeCP2-308 male and female mice share some brain metabolic features, as detected by ^1^H MRS [20]. Specifically, similarly to what was observed in MeCP2-308 males [20], we detected in the striatum of RTT female mice decreased levels of creatine and taurine, metabolites known to be involved in neuronal bioenergetics [37–39] and in the prevention from oxidative stress caused by mitochondria [40, 41]. This is consistent with emerging data demonstrating an impairment in brain mitochondria functionality and a major role of mitochondrial oxidative brain damage in RTT [42–44]. Taken together, these results suggest that MRS can provide biologically relevant measures of bioenergetic alterations in the brain, even when the size effect is relatively small.

It is however worth noting that the brain metabolic profile of RTT female mice we uncovered in the present study does not completely overlap with the one previously reported for males [20]: no changes were in fact found as for myo-inositol, an astrocyte marker, in RTT female mouse brain. Interestingly, previous data have demonstrated that MeCP2 differentially regulates the expression of GFAP, an astrocytic marker in the developing female and male brains [45]. Taken together, these results suggest caution when dealing with gender issues and further stress the need for studies focussing on the appropriate genetic and hormonal milieu in preclinical studies [46].

Another interesting finding concerns the absence of metabolic changes in the hippocampus of RTT female mice, which recapitulates previous data in males [20]. It should in fact be noted that this brain area is one of the most studied brain regions in RTT preclinical research [47–51]. These results, together with previous data in patients [52–54] and in mouse models [55, 56], point to a crucial involvement of striatal dysfunction in RTT pathogenesis, possibly explaining the wide variety of extrapyramidal symptoms found in RTT patients [57].

## 5. Conclusions

Despite the high relevance of motor dysfunction in RTT, only spare attention has so far been paid to the variety of fine motor phenotype in RTT mouse models, in particular the fine coordination skills [21, 22]. The results presented here highlight for the first time the deficient purposeful use of forepaws in MeCP2-308 female mice. Indeed, this model appears to recapitulate most of the RTT-related alterations in motor function and show some of the brain metabolic features found in patients. As a whole, results presented here provide to RTT preclinical research several innovative markers of treatment efficacy to be exploited.

## Supplementary Material

Supplementary Video 1: Illustrative portion of one experimental session of the Morag test showing the performance of a wild-type mouse.
Supplementary Video 2: Illustrative portion of one experimental session of the Morag test showing the performance of a RTT mouse.
